# Biomechanical dysregulation of SGK-1 dependent aortic pathologic markers in hypertension

**DOI:** 10.3389/fcvm.2024.1359734

**Published:** 2024-06-06

**Authors:** J. Ryan Gedney, Victoria Mattia, Mario Figueroa, Christian Barksdale, Ethan Fannin, Jonah Silverman, Ying Xiong, Rupak Mukherjee, Jeffrey A. Jones, Jean Marie Ruddy

**Affiliations:** ^1^ Division of Vascular Surgery, Medical University of South Carolina, Charleston, SC, United States; ^2^ Division of Cardiothoracic Surgery, Medical University of South Carolina, Charleston, SC, United States; ^3^ Ralph H Johnson Veterans Affairs Healthcare System, Charleston, SC, United States

**Keywords:** hypertension, aortic stiffness, SGK-1, interleukin-6, cathepsins

## Abstract

**Introduction:**

In hypertension (HTN), biomechanical stress may drive matrix remodeling through dysfunctional VSMC activity. Prior evidence has indicated VSMC tension-induced signaling through the serum and glucocorticoid inducible kinase-1 (SGK-1) can impact cytokine abundance. Here, we hypothesize that SGK-1 impacts production of additional aortic pathologic markers (APMs) representing VSMC dysfunction in HTN.

**Methods:**

Aortic VSMC expression of APMs was quantified by QPCR in cyclic biaxial stretch (Stretch) +/− AngiotensinII (AngII). APMs were selected to represent VSMC dedifferentiated transcriptional activity, specifically Interleukin-6 (IL-6), Cathepsin S (CtsS), Cystatin C (CysC), Osteoprotegerin (OPG), and Tenascin C (TNC). To further assess the effect of tension alone, abdominal aortic rings from C57Bl/6 WT mice were held in a myograph at experimentally derived optimal tension (OT) or OT + 30% +/−AngII. Dependence on SGK-1 was assessed by treating with EMD638683 (SGK-1 inhibitor) and APMs were measured by QPCR. Then, WT and smooth muscle cell specific SGK-1 heterozygous knockout (SMC-SGK-1KO^+/−^) mice had AngII-induced HTN. Systolic blood pressure and mechanical stress parameters were assessed on Day 0 and Day 21. Plasma was analyzed by ELISA to quantify APMs. Statistical analysis was performed by ANOVA.

**Results:**

In cultured aortic VSMCs, expression of all APMs was increased in response to biomechanical stimuli (Stretch +/−AngII,). Integrating the matrix contribution to signal transduction in the aortic rings led to IL-6 and CysC demonstrating SGK-1 dependence in response to elevated tension and interactive effect with concurrent AngII stimulation. CtsS and TNC, on the other hand, primarily responded to AngII, and OPG expression was unaffected in aortic ring experimentation. Both mouse strains had >30% increase in blood pressure with AngII infusion, reduced aortic distensibility and increased PPV, indicating increased aortic stiffness. In WT + AngII mice, IL-6, CtsS, CysC, and TNC plasma levels were significantly elevated, but these APMs were unaffected by HTN in the SMC-SGK-1KO^+/−^ +AngII mice, suggesting SGK-1 plays a major role in VSMC biomechanical signaling to promote dysfunctional production of selected APMs.

**Conclusion:**

In HTN, changes in the plasma levels of markers associated with aortic matrix homeostasis can reflect remodeling driven by mechanobiologic signaling in dysfunctional VSMCs, potentially through the activity of SGK-1. Further defining these pathways may identify therapeutic targets to reduce cardiovascular morbidity and mortality.

## Introduction

Hypertension (HTN) is an independent risk factor for cardiovascular morbidity and mortality (1). Maladaptive remodeling of the aortic medial extracellular matrix (ECM) under hypertensive mechanical force is driven by local production of effector proteases (2). Similar to other vascular pathologies such as aneurysmal degeneration, specifically targeting the proteolytic process to reverse or abrogate pathologic matrilysis has been elusive, but there may be an opportunity to impact tension-induced signaling in the aortic vascular smooth muscle cells (VSMCs) and directly address this detrimental remodeling.

As a foundation for this work, our laboratory has investigated the serum and glucocorticoid inducible kinase-1 (SGK-1), a signaling molecule previously associated with stress-induced signaling in other vascular beds (3, 4). Pro-inflammatory cytokine production stimulated by biaxial stretch of aortic VSMCs was abrogated by the specific SGK-1 inhibitor EMD638683 as well as by silencing RNA (5). Then, in AngII-induced HTN, the SGK-1 inhibition reduced systemic as well as local signs of inflammation despite no change in systolic blood pressure (SBP) (5). SGK-1 represents a prime candidate to transduce hypertensive aortic mechanical strain to promote VSMC transcriptional activity. Therefore advances in medical therapy of HTN must consider the intrinsic VSMC response to strain and target those alterations in signaling that impact matrix remodeling.

Many studies have described plasma biomarkers in cardiovascular disease (6), particularly those focused on aortic pathology (7–10), but have failed to operationalize this peptide analysis into clinical care. Attempts to apply single biomarker levels across a diverse population has likely contributed to these obstacles, and in this project we proposed that VSMC transcriptional activity evolves in hypertensive aortic remodeling, therefore assessing a panel of biomarkers will be more effective. By focusing on previously documented markers of dedifferentiated, dysfunctional aortic VSMCs such as interleukin-6, IL-6 (5, 11), cathepsin S, CtsS (12, 13), cystatin C, CysC (12, 14), osteoprotegerin, OPG (15, 16), and tenascin-C, TNC (17, 18), this project has utilized a murine HTN model to investigate aortic pathologic marker (APM) production, particularly as it relates to SGK-1. Further, these APMs have been assessed in parallel with ultrasound-derived mechanical aortic forces to address the hypothesis that HTN can activate SGK-1 to alter VSMC transcriptional activity to promote APM production. Demonstrating that the mechanosensitive activity of SGK-1 can impact APMs has supported this kinase as a druggable target to complement current HTN therapy to reduce cardiovascular morbidity and mortality.

## Methods

### Animal care and use

All animal care and surgical procedures were approved by the Medical University of South Carolina Institutional Animal Care and Use Committee (AR#2020-01052). Wild-type (WT) mice of the C57Bl/6J strain were purchased from Jackson Laboratories (#000664; Bar Harbor, ME) and a local breeding colony was established to support experimentation. To create a heterozygous smooth muscle specific SGK-1 knockout strain (SMC-SGK-1KO^+/−^), MyH11^Cre−EGFP^ mice were purchased from Jackson Laboratories (#007742; Bar Harbor, ME). We also acquired the SGK-1^flox/flox^ strain from the Fejes-Toth laboratory (19). Each transgenic strain had been established on C57Bl/6J background and local breeding colonies were established. Cross-breeding was completed to achieve the SMC-SGK-1KO^+/−^ mice with confirmation of genotyping through Transnetyx Inc, (Cordova, TN). Reduction in SGK-1 abundance in the murine aorta of SMC-SGK-1KO^+/−^ mice was confirmed by immunoblot using an antibody for SGK-1 (ab32374, AbCam, Waltham, MA; 1:1000; Supplementary Figure S1). Due to reports of severe physiologic impairment and potential lethality (20, 21), we have not bred SMC-SGK-1KO^−/−^ mice for experimentation. Mice were randomly assigned to treatment groups in equal proportion by sex and age. Our prior experience with IL-6 in this model of murine HTN informed the experimental grouping.

### VSMC culture and biaxial cyclic stretch with AngII stimulation

Primary VSMC lines from C57Bl/6 aortic biopsies were established using an accepted culture technique as previously described (5, 22). Isolated VSMCs were maintained in SMC specific growth media with added supplement pack (SMC Growth Medium 2; C-22062, PromoCell, Heidelberg, Germany) at 37°C in 5% CO_2_. Confluent C57Bl/6J VSMCs from culture passages 2–10 were seeded at a density of 5000 cells per cm^2^ into amino coated Bioflex-6 well plates (BF-3001A; Flexcell International Corporation, Burlington NC) and allowed to adhere overnight. Complete SMC medium was replaced with serum free SMC basal media (SMC Basal Medium 2; C-22262, PromoCell. Heidelberg, Germany) and allowed to rest for 6 h at 37°C in 5% CO_2_. Following this incubation, plates were held under Static conditions or subjected to 12% biaxial cyclic Stretch for 12 h utilizing the Flexcell culture system (Flexcell International Corporation, Burlington, NC). Separate replicates were treated with 100nM AngII. Cells were harvested to quantify expression of IL-6, CtsS, CysC, OPG, and TNC by quantitative polymerase chain reaction (QPCR).

### *Ex vivo* tension application

Following induction of anesthesia using 3% isoflurane, C57Bl/6J mice (12–20 weeks of age) underwent midline laparotomy. The abdominal aorta was harvested from the left renal vein to the bifurcation and washed in Krebs-Henseleit (K-H) buffer solution while the endothelium was bluntly denuded with the rough surface of a forceps. Aortic rings were suspended on parallel wires in the Radnoti e*x vivo* tissue myograph system with oxygenated K-H physiologic salt solution. The experimental process for defining optimal tension (OT) has been previously described by our laboratory for the murine aorta (11, 23, 24). Briefly, incremental stress-relaxation of the C57Bl/6J abdominal aortic rings was completed until the relaxation component was consistently below 10%, indicating the threshold for matrix compliance. Then the aortic rings were equilibrated at incremental tension values and treated with potassium chloride to stimulate contraction. The intersection point of compliance and maximal contractility was defined as the OT for the C57Bl/6J mouse abdominal aorta.

To assess tension-induced alterations in VSMC transcription with the integration of matrix forces, aortic rings were harvested as above and allowed 30 min of equilibration at 0 g tension in the Radnoti myograph with oxygenated K-H. Then, aortic rings were maintained at either experimentally derived OT(1.5 g) or 30% elevated tension (OT + 30%, 1.95 g) for 6 h with K-H refreshed every 30 min. Separate replicates of aortic rings were treated with AngII (100 nM) and/or the SGK-1 specific inhibitor EMD638683 (10 μM). Aortic rings were homogenized for gene expression analysis by QPCR.

### RNA extraction and quantitative polymerase chain reaction (QPCR)

Following VSMC treatment on the Flexcell system, total RNA was extracted using TRIzol Reagent (15596026, Thermo Fisher Scientific, Waltham, MA). One microgram of total RNA, determined by NanoDrop 2000 (Thermo Fisher Scientific, Waltham, MA), was reverse transcribed and converted to complementary DNA (cDNA) using the iScript cDNA synthesis kit (1708891, Bio-Rad, Hercules, CA).

Following *ex vivo* tension application on aortic rings, total RNA was extracted using Qiagen RNeasy Protect Mini Kit (74124, Qiagen, Hilden, Germany). Approximately 300 ng of total RNA, determined by NanoDrop 2000 (ThermoFisher Scientific, Waltham, MA), was reverse transcribed and converted to complementary DNA (cDNA) using the iScript cDNA synthesis kit (1708891, Bio-Rad, Hercules, CA).

Each cDNA sample was amplified with messenger RNA (mRNA) specific TaqMan Gene Expression Assays (IL-6, Mm00446190_m1; CtsS; Mm01255859_m1; CysC, Mm00438347_m1; OPG, Mm00435451_m1; TNC; Mm00495662_m1, GAPDH, Mm99999915_g1; ThermoFisher Scientific, Waltham, MA) on a CFX-96 real-time PCR machine (Bio-Rad, Hercules, CA). The relative expression of each mRNA was calculated and normalized to the expression of the house-keeping gene GAPDH. Expression values were calculated as 2^^−ΔCT^, in which the change in cycle threshold was defined as normalized gene expression.

### Immunoblot for SGK-1 and pSGK-1 in aortic rings

Relative abundance of SGK-1, phosphorylated (activated) SGK-1 (pSGK-1), and beta-actin were determined by immunoblotting as previously described (5). Briefly, following homogenization of the abdominal aortic ring, 20 µg of protein was fractionated on a 10% polyacrylamide gel by electrophoresis. The proteins were transferred to nitrocellulose membranes (0.45 µm; Bio-Rad, Hercules, CA) and incubated in antisera specific for SGK-1 (ab32374, AbCam, Waltham, MA; 1:1000), pSGK-1 (ab55281, AbCam, Waltham, MA; 1:1000), and beta-actin (1:500 in 5% BSA) in 0.1% Tween/TBS. A secondary peroxidase-conjugated antibody was applied (1:5000; 0.1% TBST). Signals were detected with a chemiluminescent substrate (Western Lighting Chemiluminescence Reagent Plus; PerkinElmer, San Jose, CA) and recorded on film. Band intensity was quantified using ImageJ 53C software (National Institute of Health, USA).

### AngII-induced HTN

Angiotensin II (A9525, MilliporeSigma) was dissolved in phosphate buffered saline and loaded at 1.46 mg/kg/day for 21 days via Azlet osmotic mini pump (model 1004; Durect Corporation, Cupertino, CA) for consistent agent delivery. Previous experimentation in this laboratory has demonstrated that this model of murine HTN through AngII infusion in C57Bl/6J (WT) mice of this age and regular chow diet did not develop aneurysms (5, 25). With SMC-SGK-1KO^+/−^ mice on the C57Bl/6J background, they were expected to respond similarly. Preliminary experimentation in our laboratory confirmed that these transgenic animals responded similarly to WT with regard to blood pressure response to AngII infusion and no change in aortic diameter was noted (data not shown).

Mice were anesthetized through 3% isoflurane induction. Subcutaneous injection of 0.05 mg/kg buprenorphine was administered and C57Bl/6J WT or SMC-SGK-1KO^+/−^ mice (12–20 weeks of age) underwent subcutaneous left back implantation of osmotic mini pump loaded with AngII. Following pump implantation, the incision was closed and mice were allowed to recover in a heated and oxygenated chamber before being returned to their littermates. Mice were monitored after surgery and sutures were removed 14 days after surgery.

### Blood pressure measurement

Mouse blood pressures were measured on Day 0 and Day 21 of treatment using CODA8 tail-cuff method (Kent Scientific, Torrington, CT) concurrent to ultrasound image acquisition (below). Although our laboratory has previously obtained blood pressure values with the animal acclimated and awake (5, 25, 26), the concurrent ultrasound procedure required anesthesia therefore animals were induced with 3% inhaled isoflurane and monitored with EKG tracing to modify anesthetic depth to maintain heart rate at 450–550 bpm during the SBP measurements.

### Ultrasound image acquisition and analysis

Ultrasound images were obtained on Day 0 and Day 21 of treatment using a VisualSonics Vevo 3100 ultrasound machine (FUJIFILM VisualSonics, Toronto, Canada). Mice were anesthetized using 3% isoflurane and placed supine on a warmed (42°C) monitoring board, and isoflurane was titrated to a heart rate of 450–550 bpm. B-mode ultrasound image loops, optimized to one cardiac cycle, were obtained of the abdominal aorta using a 55 MHz linear probe in a longitudinal plane through the center of the blood vessel, extending from the renal vein to the distal abdominal aorta proximal to the aortic bifurcation.

Ultrasound images were loaded into Vevo VascLAB software (FUJIFILM VisualSonics, Toronto, Canada). Vessel walls were labeled and tracked across the cardiac cycle such that calculations of radial strain, distensibility, and pulse propagation velocity were recorded. Radial strain represented the percent change in diameter between systole and diastole (27, 28). Distensibility was obtained in a similar fashion, but normalized to pulse pressure obtained during the concurrent tail-cuff quantifications (27, 29). Separately, time between pulse images of proximal and distal sections of the aorta were measured to obtain pulse propagation velocity, defined as the speed at which a pulse travels down the aortic wall. Representative measurements can be viewed in Supplementary Figure S2.

### Aortic tissue and plasma harvest

Terminal procedures were conducted at Day 21. Following induction of 3% isoflurane, laparotomy was performed and the aorta was exposed from the renal vein to the bifurcation. Terminal aortic diameter measurements were taken using calibrated digital microscopy Pax-it software (Villa Park, IL). Sternotomy allowed for direct left ventricular puncture and blood from the left ventricle was collected with an EDTA treated syringe. Blood samples were centrifuged at 2,500×g for 15 min at 4°C. Plasma was harvested and samples were stored in −80°C for analysis of the expression of IL-6, CtsS, CysC, OPG, and TNC by enzyme-linked immunosorbent assay (ELISA).

### ELISA

ELISA was used to quantify the concentration of IL-6 (ab100712; Abcam, Waltham, MA), CtsS (XPEM0701; XpressBio, Frederick MD), CysC (MSCTC0; R&D Systems, Minneapolis, MN), OPG (ELM-OPG-1; RayBiotech, Peachtree Corners, GA), and TNC (MBS8819882; MyBioSource, San Diego, CA) in murine plasma. Standard dilution and reagents were prepared in accordance to manufacturer's instructions. Absorbance at 450 nm was measured using a SpectraMax M3 microplate absorbance reading (Molecular Devices) with SoftMax Pro (Version 7.1) software. Value are reported as a fold change from the baseline mouse cohort (WT).

Collated data for each mouse is available in Supplementary Table S1.

### Statistical analysis

QPCR expression values for VSMCs and aortic rings were assessed as expression normalized to the baseline cohort employing the delta delta Ct method (Static VSMCs or WT OT aortic rings). As in other investigations into tissue kinase activity (26, 30), SGK-1 activity in the murine aortic rings was derived as the ratio of the OD-derived abundance for pSGK-1/SGK-1 for each aortic sample. After calculating the cytokine abundance utilizing the ELISA standard curve, the plasma sample from each treatment animal was compared to the normotensive WT control mice to generate a fold change from baseline physiology (replicate values/average WT value). Blood pressure and ultrasound-derived mechanical parameters were analyzed as discrete values. Data is represented as mean +/− standard error of the mean (SEM).

Normality of the data was assessed by the D'Agostino-Pearson Test for Skewness and Kurtosis and each component of the *in vivo* data was normally distributed except for systolic blood pressure. Non-normal distribution of systolic blood pressure was expected due to the induction effect in animals treated with AngII infusion. The VSMC cytokine expression data was normally distributed. In the ELISA data, CysC was normally distributed and IL-6, CtsS, OPG, and TNC became normally distributed after log10 transformation. In each data set, the groups were compared by ANOVA with interactions and *post hoc* mean separation was performed using the Sidak correction for multiple comparisons. Statistical tests were performed using GraphPad Prism (GraphPad Software; Boston, MA)) with significance established at *p* < 0.05.

## Results

### VSMC culture and biaxial cyclic stretch with AngII stimulation

**Figure 1:**
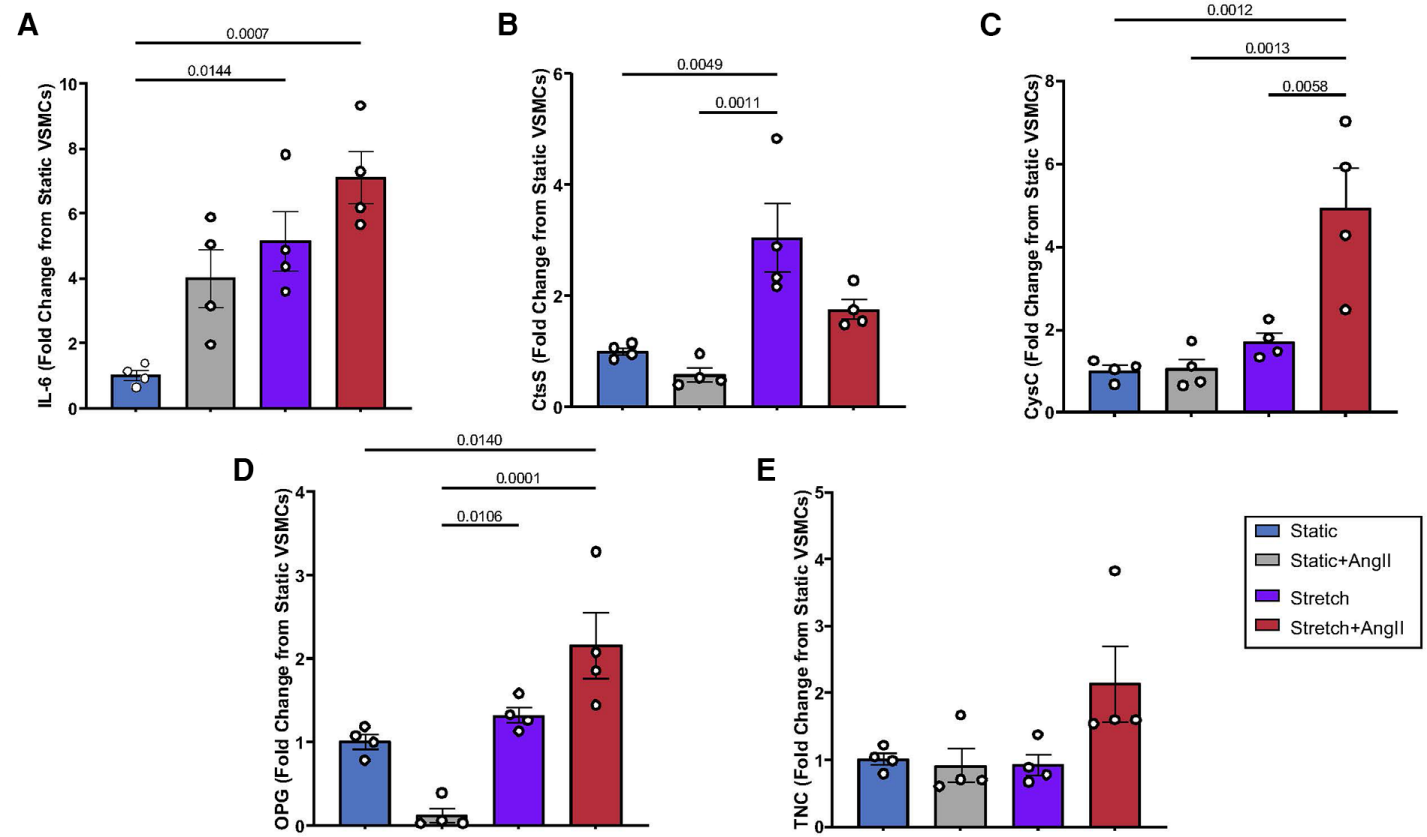
VSMC response to static vs. stretch +/− AngII for expression of (**A**) IL-6, (**B**) CtsS, (**C**) CysC, (**D**) OPG, (**E**) TNC. Expression was quantified by QPCR and data is represented as mean +/−SEM, *n* = 4 for each. Analysis included ANOVA.

Having identified commonly described cardiovascular biomarkers from literature review, this project's first undertaking was to investigate aortic VSMC expression of these APMs as they may relate to typical HTN stimuli (mechanical tension and AngII ligand). Consistent with prior work in our laboratory (5, 11), IL-6 expression was amplified by Stretch, AngII, and combined biomechanical stimulation (Figure 1A). Expression of CtsS was significantly increased by Stretch (Figure 1B), but actually returned to baseline with concurrent biomechanical stimulation, suggesting interaction with a potential negative feedback loop. CysC (Figure 1C) and OPG (Figure 1D), on the other hand, only responded to Stretch + AngII suggesting that interaction of signaling pathways was required to promote expression. Interestingly, TNC expression was nearly doubled under conditions of combined biomechanical stimulation but this was not significant (Figure 1E). While several of these peptides are known to be produced by inflammatory infiltrate, demonstrating VSMC-specific expression under standard HTN stimuli suggests that aortic VSMCs may also contribute to maladaptive remodeling.

### *Ex vivo* tension application

**Figure 2:**
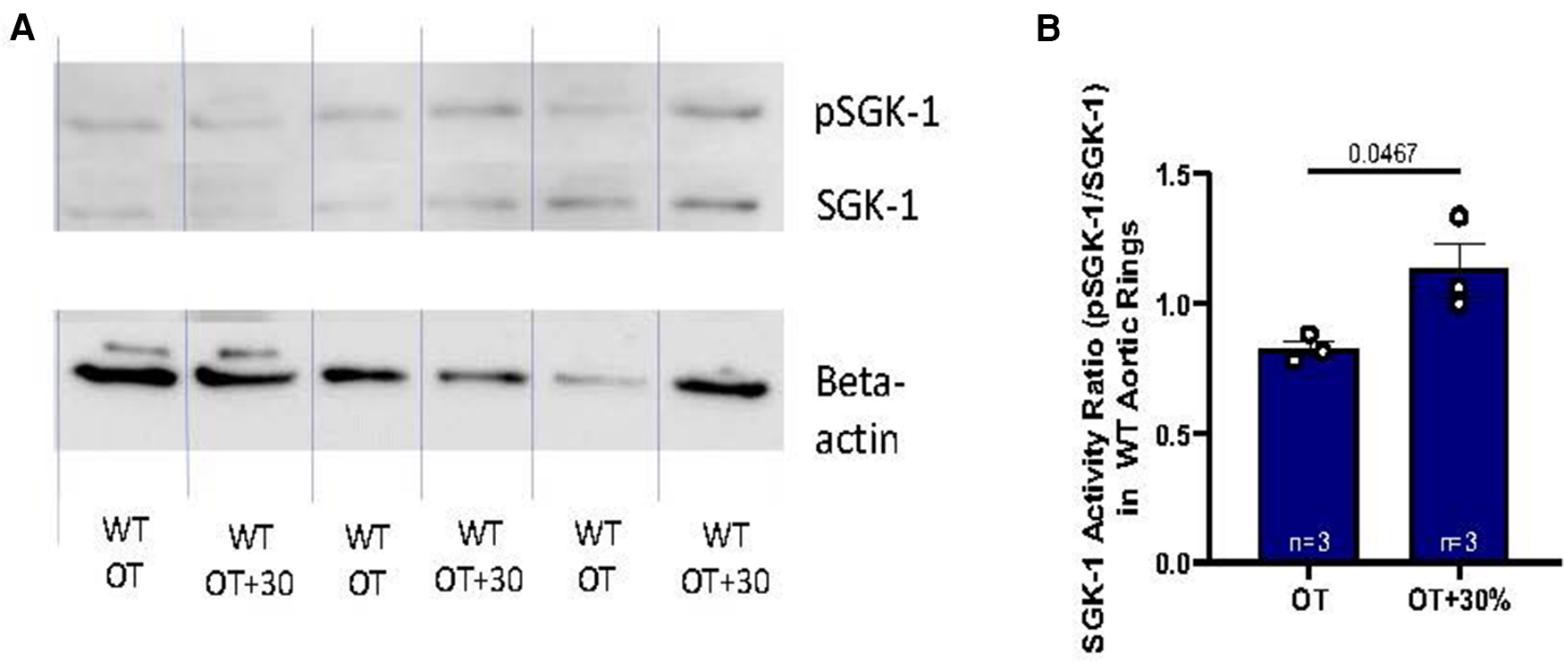
(**A**) Western blot analysis of WT abdominal aortic rings held at OT vs. OT + 30 demonstrating relative abundance of pSGK-1, SGK-1, and the housekeeping gene beta-actin. (**B**) SGK-1 activity is represented as the ratio of pSGK-1/SGK-1, *n* = 3. Data is represented as mean +/−SEM, *n* = 3. Analysis included Student's *T*-test.

The aortic medial ECM plays a major role in mechanical transduction, therefore the signaling response to elevated tension and expression of APMs were further evaluated in fresh excised murine abdominal aortic rings with particular interest in impact of SGK-1 activity. Previous work has indicated that SGK-1 can be mechanically activated in cultured aortic VSMCs (5). Here, in WT aortic rings held at 30% elevated isometric tension (OT + 30%), again the activity of SGK-1 (defined as the ratio of pSGK-1: total SGK-1) was increased (Figure 2B), indicating that this kinase may contribute to dysfunctional signaling under conditions of elevated aortic tension.

**Figure 3:**
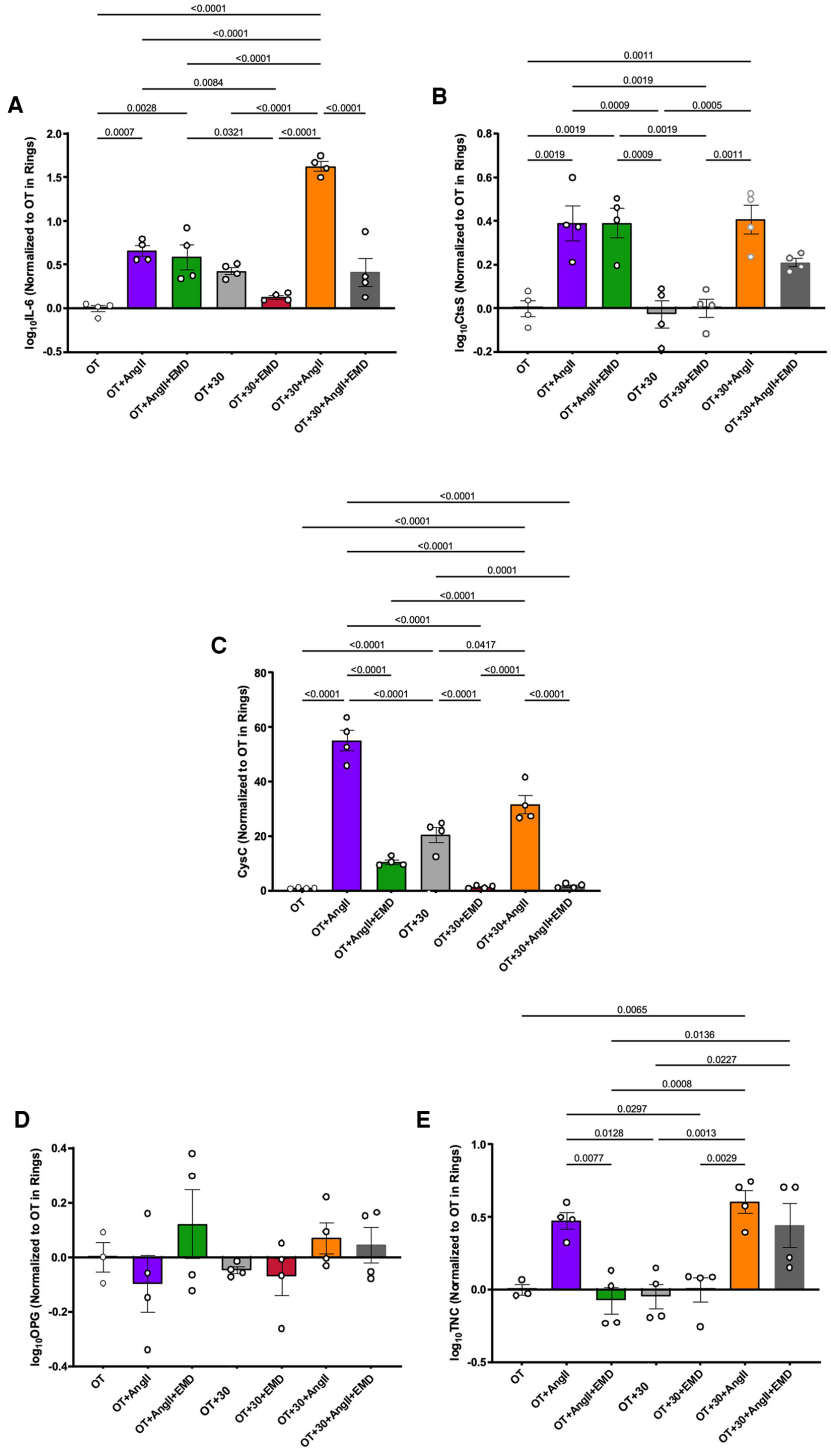
WT aortic ring response OT and OT + 30 +/− EMD and +/− AngII for expression of (**A**) IL-6, (**B**) CtsS, (**C**) CysC, (**D**) OPG, (**E**) TNC. Marker expression was quantified by QPCR and data is represented as mean +/−SEM, *n* = 4 for each. Analysis included ANOVA.

The project then explored not only how biologic and mechanical stimuli may impact APM expression, but also whether these peptides were SGK-1 dependent by treating with the specific SGK-1 inhibitor EMD638683. In aortic rings, IL-6 expression was increased by tension and continued to show SGK-1 dependence with significant inhibition by EMD treatment (Figure 3A). Interestingly, IL-6 also responded to AngII treatment and expression in aortic rings was synergistically amplified by combined biomechanical stimulation. The tension-induced expression was inhibited by EMD, confirming SGK-1 dependence. Despite the tension sensitivity demonstrated in VSMCs, CtsS expression was unchanged with tension alone in aortic rings, but significantly increased with AngII treatment and there was no additional effect with concurrent elevated tension or inhibition with EMD (Figure 3B). Tension-induced CysC expression demonstrated SGK-1 dependence in aortic rings (Figure 3C). Rather than the amplification noted in cultured VSMCs, the AngII-induced expression of CysC was abrogated by concurrent mechanical tension in aortic rings, suggesting interacting signaling pathways. This interaction is further supported by the inhibitory effect of EMD in OT + 30 as well as OT + 30 + AngII. OPG was unchanged in aortic rings treated with biomechanical stimuli (Figure 3D). Though insignificant in the cultured VSMCs, the expression of TNC in aortic rings was significantly increased by AngII with no additional effect of concurrent mechanical tension or inhibition with EMD (Figure 3E). This ring myograph system has effectively demonstrated the importance of ECM in mechanical signal transduction in the abdominal aorta with significant impact on VSMC transcriptional activity.

### AngII-induced HTN

**Figure 4:**
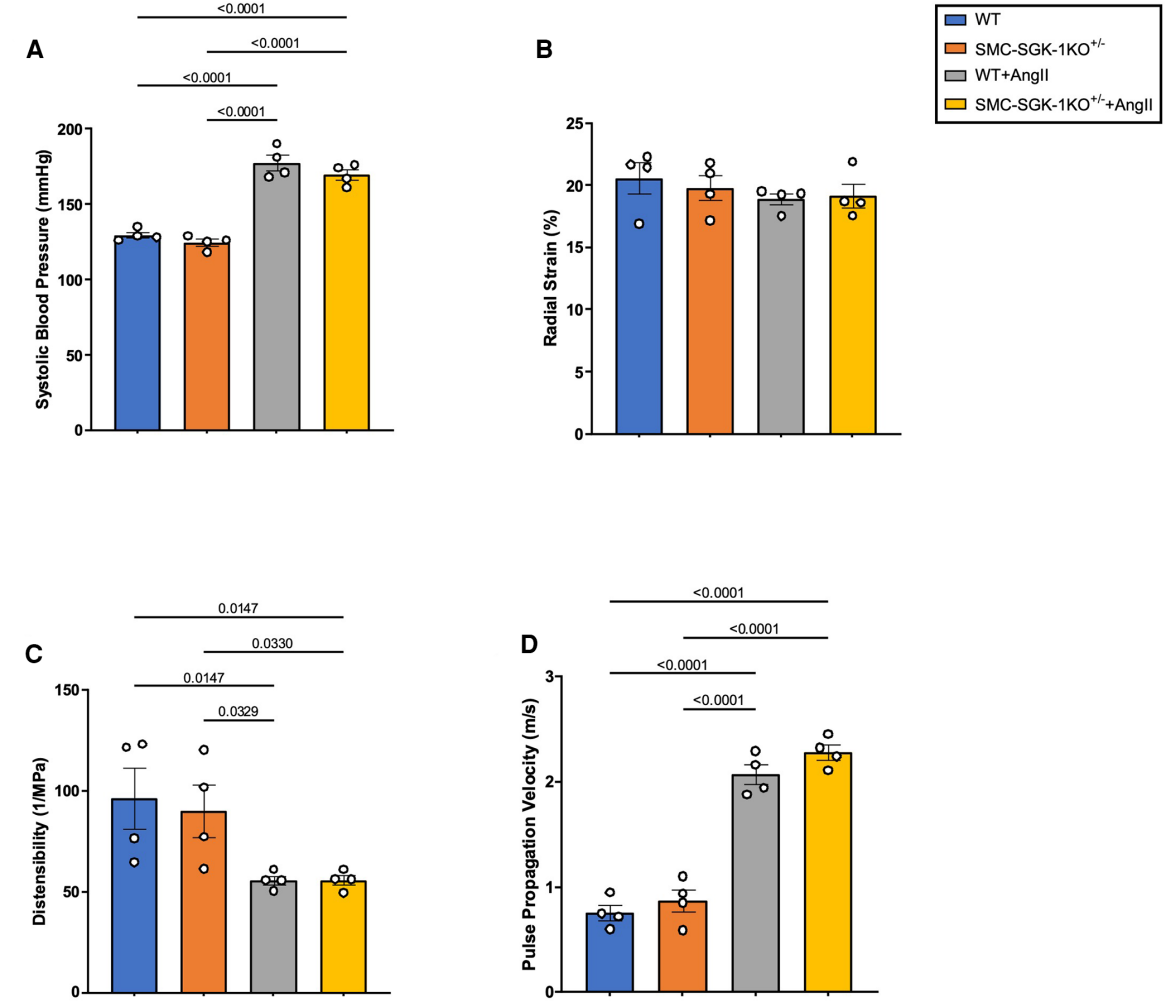
Quantification of (**A**) systolic blood pressure, (**B**) radial strain, (**C**) distensibility, and (**D**) pulse propagation velocity in WT and SMC-SGK-1KO^+/−^ mice +/− AngII infusion. Data is represented as mean +/−SEM, *n* = 4 for each. Analysis included ANOVA.

Investigating whether these biomechanical alterations in VSMC synthetic activity may be sustained in HTN and detected “remotely” in the plasma required expanding into the AngII-induced murine model of HTN. Since the *ex vivo* experimentation indicated that two of the APMs were SGK-1 dependent, the SMC-SGK-1KO^+/−^ mice had HTN induction in parallel to C57Bl/6J WT mice. At day 21, both mouse strains developed elevated systolic blood pressure and the incremental increase was similar between the strains (Figure 4A). Radial strain was unchanged (Figure 4B), but distensibility was significantly reduced (Figure 4C) and pulse propagation velocity was significantly elevated (Figure 4D) in mice with HTN. These values for distensibility and pulse propagation velocity indicate increased aortic stiffness in AngII-induced HTN. It is also important to note that the incremental change in distensibility and pulse propagation velocity in response to elevated systolic blood pressure were comparable in WT and SMC-SGK-1KO^+/−^ mice, indicating that the reduced SGK-1 abundance did not prevent the mechanical adaptation to HTN.

**Figure 5:**
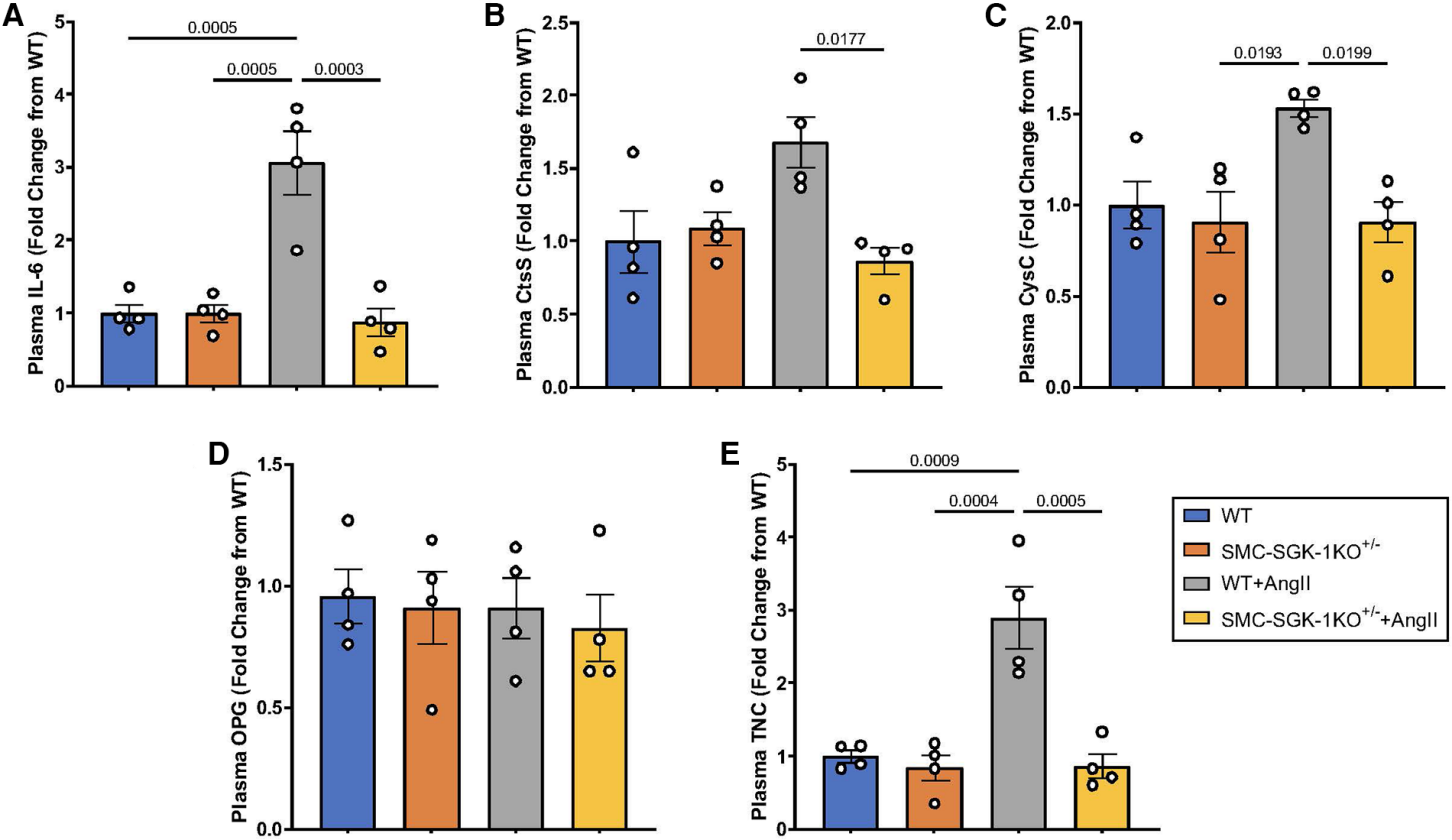
Fold change in plasma abundance of (**A**) IL-6, (**B**) CtsS, (**C**) CysC, (**D**) OPG, and (**E**) TNC in WT mice +/−AngII and SMC-SGK-1KO^+/−^ mice +/−AngII. Marker abundance was quantified by ELISA and data is represented as mean +/−SEM, *n* = 4 for each. Analysis included ANOVA.

Having quantified some of the biomechanical forces impacting the aortic wall in HTN, we were interested in whether these coincided with elevation of plasma levels of the selected APMs. Similar to the parallelism seen in the mechanical response to HTN in WT and SMC-SGK-1KO^+/−^ mice, again the baseline values of plasma APMs were analogous in both mouse strains. Then, IL-6, CysC, and TNC were elevated in WT + AngII mice, but this peptide production was abrogated in the SMC-SGK-1KO^+/−^ +AngII mice (Figures 5A, C, E). Interestingly, CtsS levels in WT + AngII mice were not elevated, but were significantly reduced in the SMC-SGK-1KO^+/−^ +AngII mice (Figure 5B). OPG was unchanged in HTN in both mouse strains (Figure 5D). Considering the systemic impact of HTN, a subset of APMs can be detected in the plasma and appear to demonstrate SGK-1 dependence.

## Discussion

Vascular remodeling in HTN is known to contribute to cardiovascular events, but clinical management of blood pressure has been unable to prevent the morbidity related to aortic stiffness and microvascular end organ damage. Identification of novel molecular targets to reduce matrix pathology may complement anti-hypertensive initiatives and reduce cardiovascular events. As single cell RNA inquiries, protein chip analyses, and lineage tracing techniques have identified clusters of genes upregulated within VSMC sub-populations in vascular pathology (12, 17, 31), this investigation has utilized that literature to focus on well-known aortic pathology markers that may be tracked in the blood stream to represent the aortic VSMC stress response. By employing cell culture, isolated aortic rings, and a validated model of murine HTN, this project aimed to define how physiologically relevant biomechanical stimuli altered aortic VSMC transcriptional activity. Markers consistent with inflammation, matrilysis, and fibrosis were elevated in this investigation and demonstrated dependence on signaling through the mechanosensitive kinase SGK-1. Refining the role of SGK-1 in VSMC synthetic activity may support this kinase as a viable target to improve vascular health in patients with HTN to reduce cardiovascular events.

HTN has been described as a pro-inflammatory state, with IL-6 being a major contributor (32, 33). Several *in vitro* and *ex vivo* investigations have reported that hypertensive stimuli such as mechanical tension and the AngII ligand can promote VSMC production of IL-6 (11, 34). The synergistic interactions identified in the current work supports complementary intracellular pathways as proposed in prior computational analysis wherein SGK-1 was a node in three positive feedback loops promoting IL-6 expression in VSMCs (5). The elevated circulating IL-6 levels have been suspected to drive the inflammatory cell infiltration as mediators of proteolytic activity (5, 35), but this project supports the concept that native VSMCs can shift toward inflammatory transcriptional activity to produce IL-6 and additional proteolytic/matrilytic genes can be expressed as well. In this way, IL-6 is unlikely to represent a therapeutic target for patients with HTN, but may be developed into a functional biomarker to gauge the inflammatory state and response to therapy.

Prior work in our laboratory utilizing this murine HTN model failed to identify increased expression or abundance of major matrix metalloproteinases in the murine abdominal aorta, but the cysteine protease system was responsive (25). In addition to its activity within lysosomes of VSMCs and inflammatory cells, CtsS has been linked to lysis of ECM in abdominal aortic aneurysm (36), intimal hyperplasia (37), and pulmonary artery hypertension (38). Regarding the VSMC synthetic activity under HTN biomechanical conditions assessed in this project, CtsS carries particular relevance because it represents a macrophage-like phenotype switch that has been observed in other vascular pathology (12), and can promote ECM degradation with loss of VSMC adhesion points that also contributes to phenotype switching (39). In fact, the environment of VSMCs on culture plate vs. *ex vivo* with maintained ECM (as in aortic ring) has been previously shown to impact synthetic activity (40), and likely impacted the results here as well. CtsS expression responded to Stretch as well as combined Stretch + AngII in cultured murine aortic VSMCs, but was not mechanosensitive in aortic rings with intact ECM. In the *ex vivo* system, increased CtsS expression was only achieved through AngII ligand-dependent signaling, which carries significant implications for further application to vascular disease. In this model of murine HTN, CtsS production was prevented in SMC-SGK-1KO^+/−^ +AngII mice, suggesting that the reduced availability of SGK-1 impacted the downstream intracellular signaling of AngII as it impacted CtsS production. While post-transcriptional and post-translational modifications may play a role in the delivery of CtsS locally and systemically in HTN, it is also important to consider how AngII and SGK-1 signaling pathways may overlap/intersect to influence synthetic activity in VSMCs under biomechanical stress. The existence of complementary effects would greatly emphasize the benefit of SGK-1 as a therapeutic target, possibly in conjunction with AngII modifying strategies, and warrants further investigation.

Coexistent to the body of work supporting CtsS as a plasma biomarker for several cardiovascular disease states (41–43), there are many reports indicating value in quantifying CtsS in tandem with its major inhibitor CysC (14, 44). Likewise, as an independent biomarker, CysC has been associated with cardiovascular mortality and peripheral arterial disease severity (45, 46). Utilizing the biomechanical stimuli described in this experimentation, cultured VSMCs, aortic ring tissue, and plasma from WT + AngII mice demonstrated CysC response to tension and/or AngII which was dependent on SGK-1. In advanced vascular disease processes, tracking CysC vs. CtsS to generate an activity ratio has been proposed, with CtsS remaining elevated and CysC demonstrating a deficit (44). This investigation noted aortic and plasma levels of these markers are both elevated in short term HTN, indicating that levels in chronic HTN warrants additional investigation. Overall, since CtsS and CysC responded to AngII treatment *ex vivo* and in HTN, exploring opportunities to modulate AngII ligand dependent signaling and SGK-1 activity through pharmacologic targeting may represent a meaningful way to shift matrilysis.

On the other hand, should therapeutic focus prioritize promoting healthy matrix deposition? The matricellular glycoprotein TNC plays a significant role in blood vessel development during embryonic stages and can be upregulated in wound healing, but has low expression in adult healthy vasculature (47, 48). Pathologic increases in VSMC production of TNC have been detected in intimal hyperplasia following revascularization (49, 50), and the inflammatory infiltrate in aneurysm disease has been suspected to contribute to the elevation of TNC in that tissue (51). The role of TNC in cardiovascular disease remains poorly understood due to contradictory reports of detriment vs. benefit of the TNC-dependent remodeling, particularly regarding aortic response to AngII signaling (18, 52). Alternatively, systemically detected TNC may represent peptide released from damaged vasculature, aligning with other reports promoting TNC as a biomarker with prognostic relevance in cardiovascular injury and acute aortic pathology (53, 54). In the experimentation presented here, TNC expression was upregulated by AngII signaling in cultured VSMCs, aortic rings, and WT mice with HTN. Interestingly, similar to CtsS, TNC plasma values were reduced in the SMC-SGK-1KO^+/−^ +AngII mice, suggesting that SGK-1 may contribute to this signaling cascade. With CtsS and TNC representing different aspects of VSMC transcriptional activity toward matrix integrity, this also raises the question of how SGK-1 may participate in defining the VSMC response to biomechanical stimuli, and therefore how targeting SGK-1 may promote vascular health.

### Limitations

While this project sought to assess production of APMs with biomechanical stimuli under complementary experimental conditions, there are several limitations. Aortic ring analysis enables examination of the aortic wall response to tension in the absence of inflammatory cell infiltrate, but does not entirely isolate VSMCs. The endothelium is removed in vessel preparation but contribution of native fibroblasts or resident macrophages may also impact our quantification of gene expression. Future directions of this experimentation will include spatial analysis to colocalize relevant biomarkers to cells of the aortic wall, with a focus on more directly addressing characterizing the VSMC phenotypes in hypertensive aortic remodeling. Also, while the magnitude of mechanical force on the aortic wall was confirmed to be consistent among the WT and SMC-SGK-1KO^+/−^ mice at baseline and when HTN was induced, this experimentation did not explore changes with anti-hypertensive treatment. These drug therapies may impact mechanical forces as well as vasoactive peptide signaling cascades and will be explored in future directions of this investigation. Regarding sample variation among the hypertensive mice, this phenomenon may reduce statistical relationships, but also demonstrates the potential difficulties with translation to clinical application in large populations, thereby refining the selection process for future biomarker studies. The choice of a single timepoint also impacts the results reported here. At 21 days, mice have stable HTN and prior work in our lab has demonstrated this to be adequate time for inflammatory cell infiltrate (5), thereby suggesting that physiologically and pathologically relevant mechanisms have begun. However, extending this experimental period will be vital to understanding the effects of chronic HTN and addressed in upcoming studies.

## Conclusion

In HTN, changes in the plasma levels of markers associated with matrix homeostasis can reflect remodeling driven by mechanobiologic signaling in dysfunctional VSMCs, potentially through the activity of SGK-1. Further defining these pathways and may identify therapeutic targets to reduce cardiovascular morbidity and mortality.

## Data Availability

The raw data supporting the conclusions of this article will be made available by the authors, without undue reservation.

## Supplementary material

Supplementary Figure S1 Representative image of relative abundance of SGK-1 (normalized to *α*-tubulin) in C57Bl/6J, SGK-1^flox+/+^, and SMC-SGK-1KO^+/−^. The bar graphs represent abundance from a single aortic sample (*n* = 1) as quantified in the immunoblot segment pictured above utilizing antibody to SGK-1 (ab32374, AbCam, Waltham, MA; 1:1000).

Supplementary Figure S2 Representative images and quantifications of Radial Strain, Distensibility, and Pulse Propagation Velocity at (A) Day 0 vs. (B) Day 21 in a WT + AngII mouse.

Supplementary Table S1 Complete data for *in vivo* murine experimentation with and without AngII-induced hypertension.
