# Supplementary figures and images for: Biomechanical dysregulation of SGK-1 dependent aortic pathologic markers in hypertension
Source: Front Cardiovasc Med. 2024 Jun 6;11:1359734. doi: 10.3389/fcvm.2024.1359734 (PMC11187291; doi:10.3389/fcvm.2024.1359734)

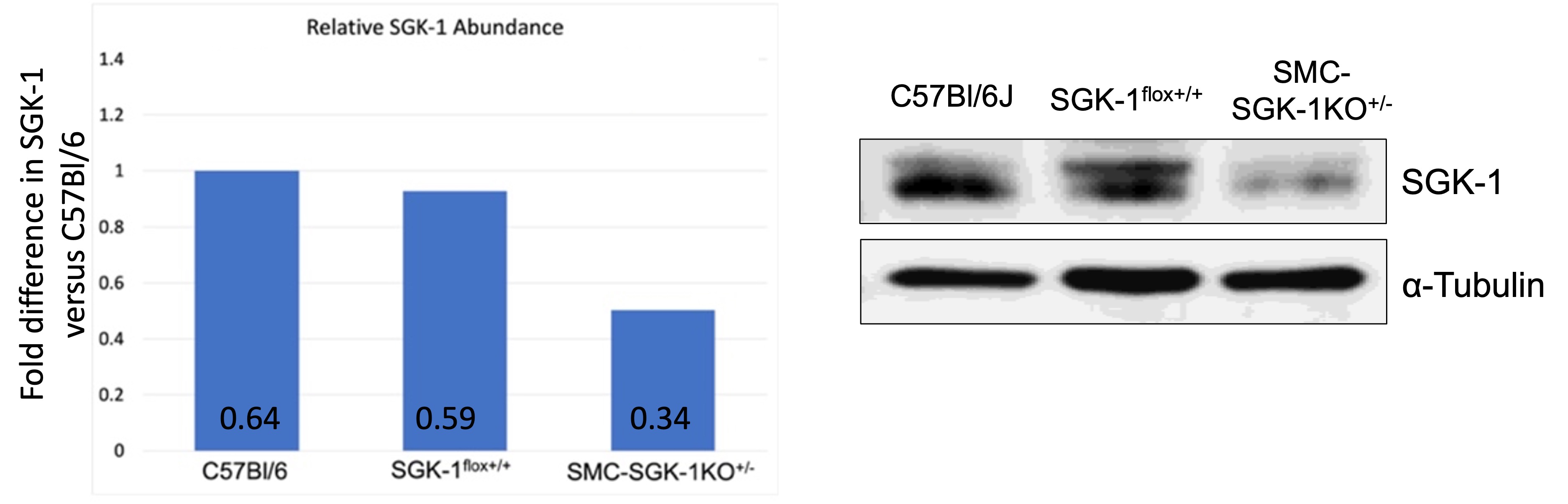

Supplement: Supplementary Figure S1 — Representative image of relative abundance of SGK-1 (normalized to α-tubulin) in C57Bl/6J, SGK-1flox+/+, and SMC-SGK-1KO+/−. The bar graphs represent abundance from a single aortic sample (n = 1) as quantified in the immunoblot segment pictured above utilizing antibody to SGK-1 (ab32374, AbCam, Waltham, MA; 1:1000). [file Image1.jpeg]

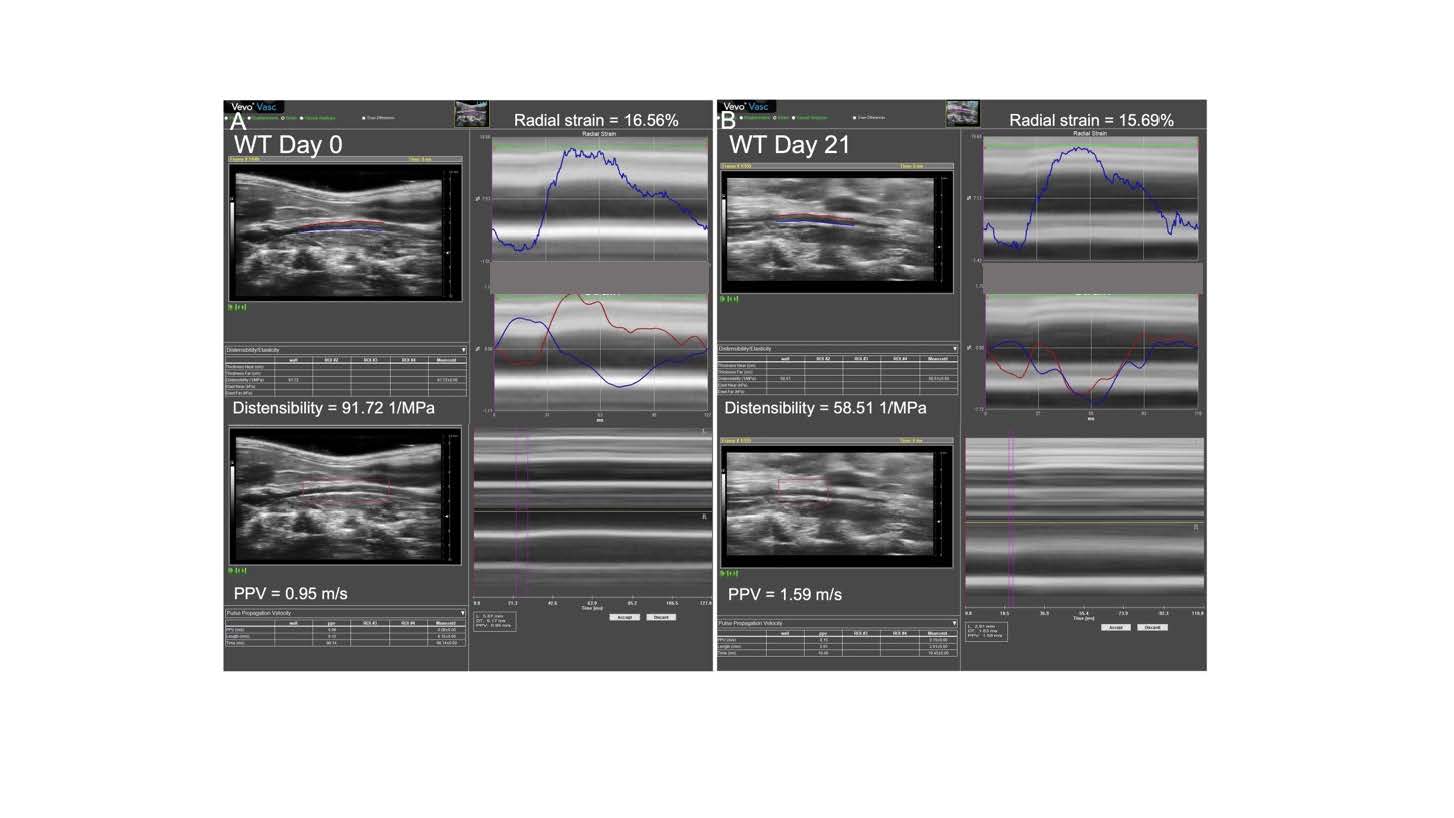

Supplement: Supplementary Figure S2 — Representative images and quantifications of Radial Strain, Distensibility, and Pulse Propagation Velocity at (A) Day 0 vs. (B) Day 21 in a WT + AngII mouse. [file Image2.jpeg]

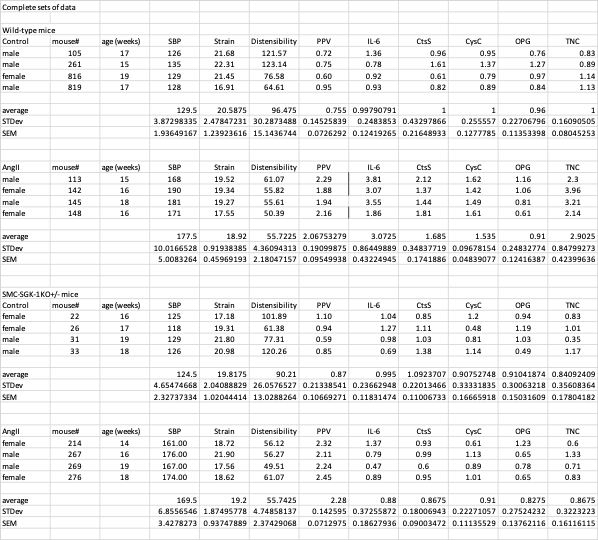

Supplement: Supplementary Table S1 — Complete data for in vivo murine experimentation with and without AngII-induced hypertension. [file Image3.png]
